# Comparison of outcomes following surgical resection, percutaneous ablation or stereotactic body radiation therapy in early‐stage, solitary and small (≤3 cm) treatment‐naïve hepatocellular carcinoma

**DOI:** 10.1002/cam4.6978

**Published:** 2024-02-24

**Authors:** A. M. Maher, M. Shanker, H. Y. H. Liu, Y. Lee, D. Leggett, P. Hodgkinson, D. Pryor, K. A. Stuart

**Affiliations:** ^1^ Gastroenterology and Hepatology Princess Alexandra Hospital Brisbane Queensland Australia; ^2^ Faculty of Medicine The University of Queensland Herston Queensland Australia; ^3^ Radiation Oncology Princess Alexandra Hospital Brisbane Queensland Australia; ^4^ Department of Medical Imaging Princess Alexandra Hospital Brisbane Queensland Australia; ^5^ Department of Hepatobiliary and Pancreatic Surgery Princess Alexandra Hospital Brisbane Queensland Australia; ^6^ Queensland Liver Transplant Service Brisbane Queensland Australia

**Keywords:** hepatocellular carcinoma (clinical), hepatocellular carcinoma (treatment), hepatology

## Abstract

**Introduction:**

Stereotactic body radiation therapy (SBRT) is associated with high local control rates in hepatocellular carcinoma (HCC). This study reports the outcomes of SBRT compared to surgical resection (SR) and percutaneous ablation (PA) for treatment‐naïve, solitary HCCs ≤3 cm.

**Methods:**

This was a retrospective study of patients with BCLC stage 0/A HCC with a single ≤3 cm lesion, treated with curative intent between 2016 and 2020. SBRT was used for patients considered unsuitable for SR or PA. The co‐primary endpoints were progression‐free survival (PFS) and overall survival (OS). The secondary endpoints were treatment‐related clinical toxicity rates and local control (LC) rates.

**Results:**

There were 112 patients included in this study. SBRT was delivered in 36 patients (32.1%), 51 had PA (45.5%) and 25 underwent SR (22.3%). Median follow‐up was 23 months (range, 3–60 months) from diagnosis. The 3‐year PFS and OS were 67% and 69% following SBRT, 55% and 80% following PA, and 85% and 100% following SR, respectively. Patients in the SR cohort had significantly better 3‐year PFS and OS compared to SBRT and PA groups (*p* = 0.03 and *p* = 0.04, respectively). There was no significant difference in PFS (*p* = 0.15) or OS (*p* = 0.23) between SBRT and PA treated patients. The 3‐year LC rate for the entire cohort was 98%.

**Conclusions:**

In patients with treatment‐naïve, early‐stage solitary HCCs ≤3 cm, SBRT was associated with comparable PFS, OS and LC outcomes to PA. SBRT should be considered as a curative intent therapy to avoid treatment stage migration in this favourable prognostic cohort of patients.

## 1 INTRODUCTION

In 2020, HCC was the sixth most diagnosed cancer and the third leading cause of cancer‐related deaths worldwide.^1^ In Australia, HCC prevalence has increased over the last four decades, with the age‐standardised incidence increasing from 1.8 in 1982 to 8.6 in 2019 per 100,000.^2^ Treatment for HCC is based on accurate staging, patient performance status and liver function with multidisciplinary input. For early‐stage HCC (Barcelona Clinic Liver Cancer [BCLC] Stages 0/A), curative surgical resection (SR) is recommended,^3^ however, over 75% of patients are not suitable for surgical management.^4^, ^5^, ^6^

Percutaneous ablation (PA) is the standard curative intent therapy in early‐stage and solitary HCC ≤3 cm in non‐surgical patients.^3^ However, previous reports have suggested up to 60% of patients are unsuitable for PA, predominantly due to tumour location.^6^ These patients may subsequently be offered treatment stage migration to palliative intent therapies.^7^

Stereotactic body radiation therapy (SBRT) is an effective liver directed therapeutic option associated with high local control rates. Recent systematic reviews of SBRT in early‐stage HCC have demonstrated 3‐year local control rates of 93%.^8^ Utilisation of SBRT in HCC has also increased worldwide, with application in a range of disease stages.^9^ However, there is limited data on survival outcomes following SBRT in the management of treatment naïve, early‐stage and solitary HCCs.

We thus conducted this study to report the survival outcomes following curative intent SBRT compared to PA and SR in patients with treatment naïve, early‐stage and solitary HCCs up to 3 cm.

## 2 MATERIALS AND METHODS

### 2.1 Study population

This was a retrospective analysis of a prospectively collected database of patients with an initial diagnosis of a solitary ≤3 cm HCC presented at our HCC multidisciplinary meeting (MDM) between June 2016 and June 2020, who were treated with curative intent therapies. The diagnosis of HCC was made based on dynamic imaging characteristics according to the American Association for the Study of Liver Disease (AASLD) criteria or histopathology if imaging was not confirmatory.^3^, ^10^

The inclusion criteria were:

1. Initial diagnosis of BCLC Stage 0/A and solitary ≤3 cm HCC,
2. Child‐Pugh (CP) score ≤B7,
3. Eastern Cooperative Oncology Group (ECOG) performance status of ≤2,
4. Treated with SR, PA or SBRT with curative intent.

The exclusion criteria were:

1. Age <18 years,
2. History of prior treatment for HCC,
3. Multifocal HCC,
4. >3 cm HCC,
5. Evidence of macrovascular invasion or extrahepatic metastases.

Liver function was assessed using Child‐Pugh (CP), Model for End‐Stage Liver Disease (MELD) and Albumin‐bilirubin (ALBI) grade. Portal hypertension was reported if there was a history of ascites, varices or presence of splenomegaly with thrombocytopaenia. Patient's non‐cancer performance status was assessed using the Eastern Cooperative Oncology Group (ECOG), and Charlson Comorbidity Index (CCI) at the time of the HCC MDM.

Ethical approval for this study was granted by the Institution's Human Research Ethics Committee (HREC/11/QPAH/76) who granted a waiver for patient consent.

### 2.2 Principles of treatment determination

Patients were reviewed at our HCC MDM with a consensus recommended management plan for a curative intent therapy, namely SR, PA or SBRT. SBRT was typically recommended if the patient was unsuitable for SR and the tumour location deemed PA unsuitable (i.e. subphrenic or subcapsular location or close proximity to central vessels or biliary structures). All patients had regular follow‐up, including biochemical analysis with tumour markers and imaging. If there was evidence of recurrence, the patient was re‐presented at the MDM.

### 2.3 Stereotactic body radiation therapy

SBRT with curative intent was delivered in the outpatient setting in three or five fractions using a coplanar volumetric modulated arc technique, dependent on tumour location, liver function and proximity to organs at risk. A three‐fraction approach was preferred for patients with well‐preserved liver function (non‐cirrhotic or CP‐A5) and located away from the central liver, bowel or pericardium. We have previously described our treatment protocol including immobilisation, motion management, target volumes and prescription aims in detail.^11^

### 2.4 Percutaneous ablation

Percutaneous microwave ablation (MWA) was performed using real time imaging guidance and the Emprint™ Ablation System (Covidien‐Medtronic, Minneapolis, MN). Thermal ablation was achieved using a 2.45 GHz M system with an internal cooled saline infusion (Thermosphere™, Covidien, Boulder, CO). The energy imparted was calculated using target lesion volume, most commonly 100 watts for 10 min with subsequent ablation of the tract. During the time period of this study, most MWA patients at our centre were admitted overnight the day before (*n* = 38, 75%) and all stayed overnight after MWA. Since this study, all patients undergoing MWA are admitted on the day and discharged the following day.

### 2.5 Surgical resection

SR was performed when the HCC was confined to the liver with sufficient liver remnant to maintain robust hepatic function. Pre‐operative surgical evaluation involved assessment of the patient's age, co‐morbidities, presence/absence of portal hypertension, liver functional reserve including indocyanine green clearance (ICG) testing, anatomical location of the HCC and whether it necessitated a major liver resection (>3 segments). Patients were admitted the day of surgery. The extent of liver resection was determined by the position of the HCC. Sixteen (64%) patients underwent single segment resection, one (4%) patient had subsegmental, four (16%) patients had multiple segments and four (16%) patients had a major hemi‐hepatectomy. No patient required routine intensive care unit (ICU) admission post‐operatively. One patient was admitted to ICU for respiratory support in the setting of hospital acquired pneumonia.

### 2.6 Statistical considerations

The close‐out date for the study was 30th June 2021. The co‐primary endpoints were progression‐free survival (PFS) and overall survival (OS). The secondary endpoints were treatment‐related clinical toxicity rates, change in CP score of >1 at three and 12‐month post treatment and local control (LC) rates. Local recurrence was defined at our MDM as recurrent HCC at the surgical margins for SR, recurrence in the ablation cavity for PA and progression of the target lesion based on Response Evaluation Criteria in Solid Tumours v1.1 for SBRT. Intrahepatic recurrences beyond the definition of local recurrence were considered to be out of field, intrahepatic progression.

The Kaplan–Meier method was used to estimate PFS and OS from the date of diagnosis at MDM to any disease progression or death event for PFS and any disease event for OS. Patients were censored at time of last follow‐up or liver transplantation (LT). Treatment‐related clinical toxicities were graded using the Common Terminology Criteria for Adverse Events (CTCAE v4.03) for PA and SBRT. Post surgical complications were graded using the Clavien‐Dindo classification.

Statistical analyses were performed on SAS (Statistical Analytical Sciences Studio Release 3.7, SAS Institute INC, Cary, NC. USA).

## 3 RESULTS

### 3.1 Study cohort characteristics

**FIGURE 1:**
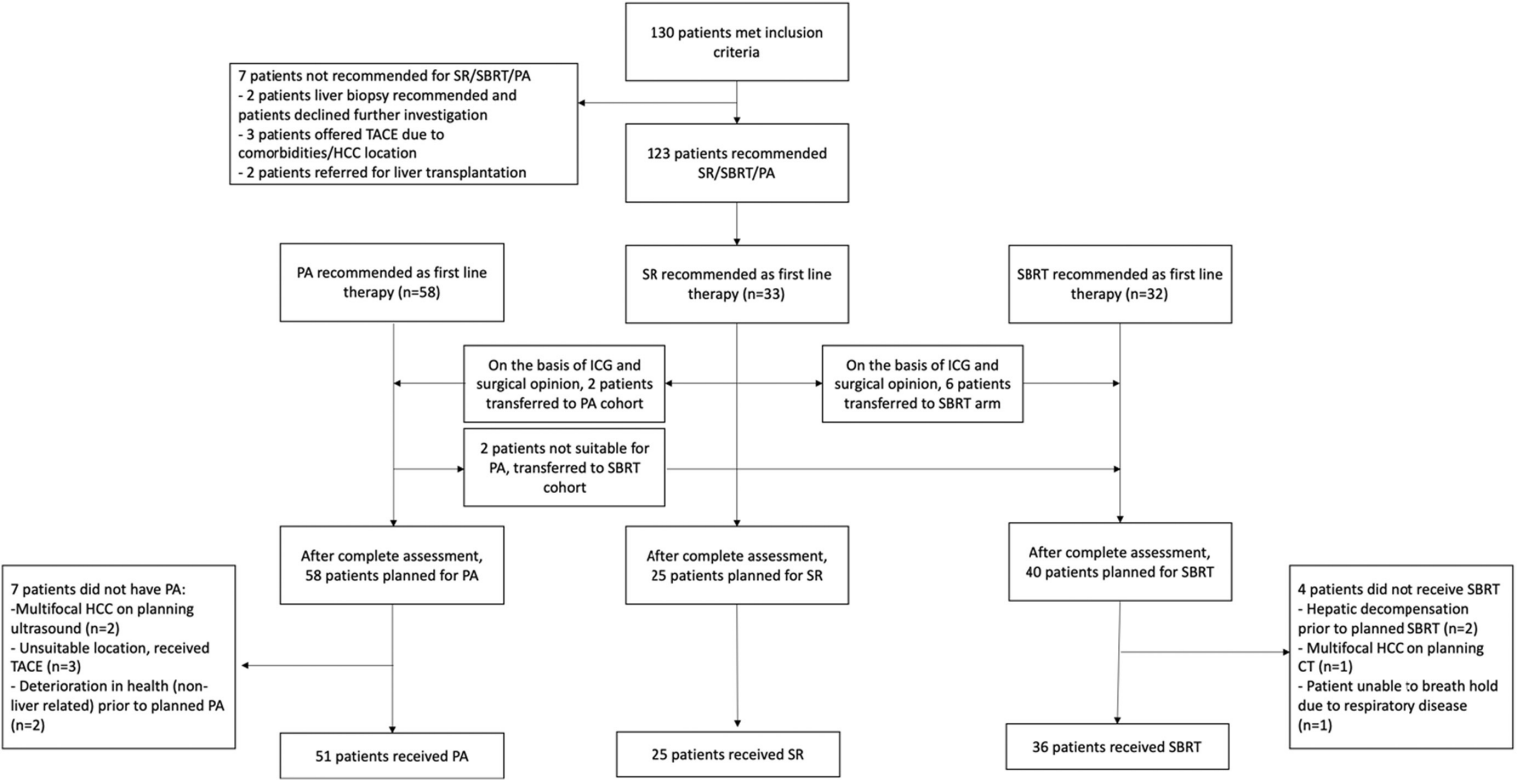
CONSORT diagram representing identification of patients suitable for study inclusion.

Two thousand three hundred fifty‐two patients were reviewed in the HCC MDM between June 2016 and June 2020. 112 patients met the study inclusion criteria and received curative intent therapies with either SBRT, PA or SR. (Figure 1) Of the 36 patients who received SBRT, reasons for not offering PA included subcapsular (*n* = 9) or subdiaphragmatic (*n* = 10) location, proximity to vessels (*n* = 5) or other structures (*n* = 7) or not visible on ultrasound (*n* = 5). These 36 patients were considered non‐surgical due to limited hepatic reserve or advanced portal hypertension (*n* = 11), patient co‐morbidities or frailty (*n* = 23), patient preference for a less invasive procedure (*n* = 1) and anatomical difficulty (*n* = 1, prior renal transplantation).

Baseline patient characteristics of the entire study cohort and per treatment were described in Table 1. The mean age of the study cohort was 64 years (SD 9 years) and the majority had liver cirrhosis (93.8%). The most common cirrhosis etiologies were hepatitis C (HCV) and metabolic associated fatty liver disease (MAFLD) (50.9% and 22.3%, respectively). The median treated HCC size was 20 mm (range 13–30 mm).

**TABLE 1:** Baseline clinical characteristics of patients.

	All (*n* = 112)	SR (*n* = 25)	PA (*n* = 51)	SBRT (*n* = 36)	*p* Value
Mean age in years (±SD)	63.9 (9.0)	60.6 (9.2)	62.6 (8.8)	68.1 (7.6)	<0.05
Number of men, *N* (%)	88 (78.6)	20 (80)	41 (80.4)	27 (75)	0.82
Primary aetiology					
MAFLD	25 (22.3)	2 (8)	11 (29.7)	12 (33.3)	<0.05
ARLD	18 (16.1)	2 (8)	7 (13.7)	9 (25)	
HCV	57 (50.9)	13 (52)	32 (62.7)	12 (33.3)	
HBV	9 (8)	7 (28)	1 (2)	1 (2.8)	
Other	3 (2.7)	1 (4)	0 (0)	1 (2.8)	
Cirrhosis co‐factor present, *N* (%)	49 (43.8)	11 (44)	26 (51)	12 (33.3)	0.06
Cirrhosis present, *N* (%)	105 (93.8)	18 (72)	51 (100)	36 (100)	<0.05
Portal hypertension present, *N* (%)	45 (40.2)	3 (12)	22 (43.1)	20 (55.6)	<0.05
Prior decompensation event, *N* (%)	12 (10.7)	1 (4)	5 (9.8)	6 (16.7)	0.28
Number of patients with CPS 5/6/7	73/19/13	18/0/0	34/12/5	21/7/8	<0.05
Median MELD score (range)	9 (6–15)	8 (6–11)	9 (6–15)	9 (6–14)	0.08
ALBI Grade 1/2, *N*	67/45	23/2	26/25	19/18	<0.05
ECOG 0/1/2 (*N*)	84/22/6	22/3/0	40/9/2	22/10/4	<0.05
Median CCI (range)	5 (2–9)	3 (2–6)	5 (3–9)	5 (3–9)	<0.05
Median serum αFP (ng/dL) (range)	5 (0.9–15,200)	5 (0.9–15,200)	5.4 (1.2–840)	4.25 (1–170)	0.15
Median HCC size in mm (range)	20 (13–30)	20 (14–30)	18 (13–25)	21 (14–30)	<0.05
BCLC Stage 0/A (*N*)	47/65	8/17	30/21	9/27	<0.05
Presence of indeterminate lesions, *N* (%)	28 (25)	4 (16)	19 (37.3)	5 (13.9)	<0.05
Median Indocyanine Green Clearance (ICG) R15 (range) and number of patients tested.	10.4 (12) *N* = 32	5.2 (3) *N* = 21	15.7 (16.7) *N* = 4	22.9 (16.5) *N* = 7	<0.05
Mean time from MDM to treatment in days (SD)	47.8 (40.2)	61.9 (50)	51.5 (39)	32.8 (29)	<0.05
Median length of inpatient stay in days (range)	3 (0–42)	6 (3–42)	3 (1–7)	0	<0.05

The 25 patients in the SR group were younger, had fewer co‐morbidities based on CCI, less likely to have advanced liver disease with 28% not having cirrhosis and only three (12%) had portal hypertension.

In the SBRT group, patients were prescribed 36–48 Gy in 3 fractions (median dose: 45 Gy) or 30–50 Gy in 5 fractions (median dose: 50 Gy). Liver mean achieved in the 3 fraction group ranged between 2.9 and 7.7 Gy (median: 5.2 Gy) and 3.1–12 Gy in the 5 fraction group (median: 5.7 Gy). Four patients had fiduciary markers inserted to assist with treatment matching in the SBRT group.

All patients in the SBRT and PA groups had cirrhosis and the two cohorts were comparable in terms of liver disease severity: median CP and ALBI grade (5 vs 5, *p* = 0.34, 1 vs 1, *p* = 0.94, respectively) and proportion with portal hypertension (55.6% vs 44.1%, *p* = 0.45). The SBRT treated patients were significantly older compared to the PA or SR cohorts (68.1 vs 62.6 years, *p* < 0.05 and 68.1 vs 60.6 years, *p* < 0.05, respectively). A greater proportion of SBRT patients had worse performance status determined by ECOG compared to the SR cohort (*p* < 0.05). There was no significant difference in ECOG (*p* = 0.11) or CCI (*p* = 0.27) between the SBRT and PA cohorts.

### 3.2 Overall survival (OS)

**FIGURE 2:**
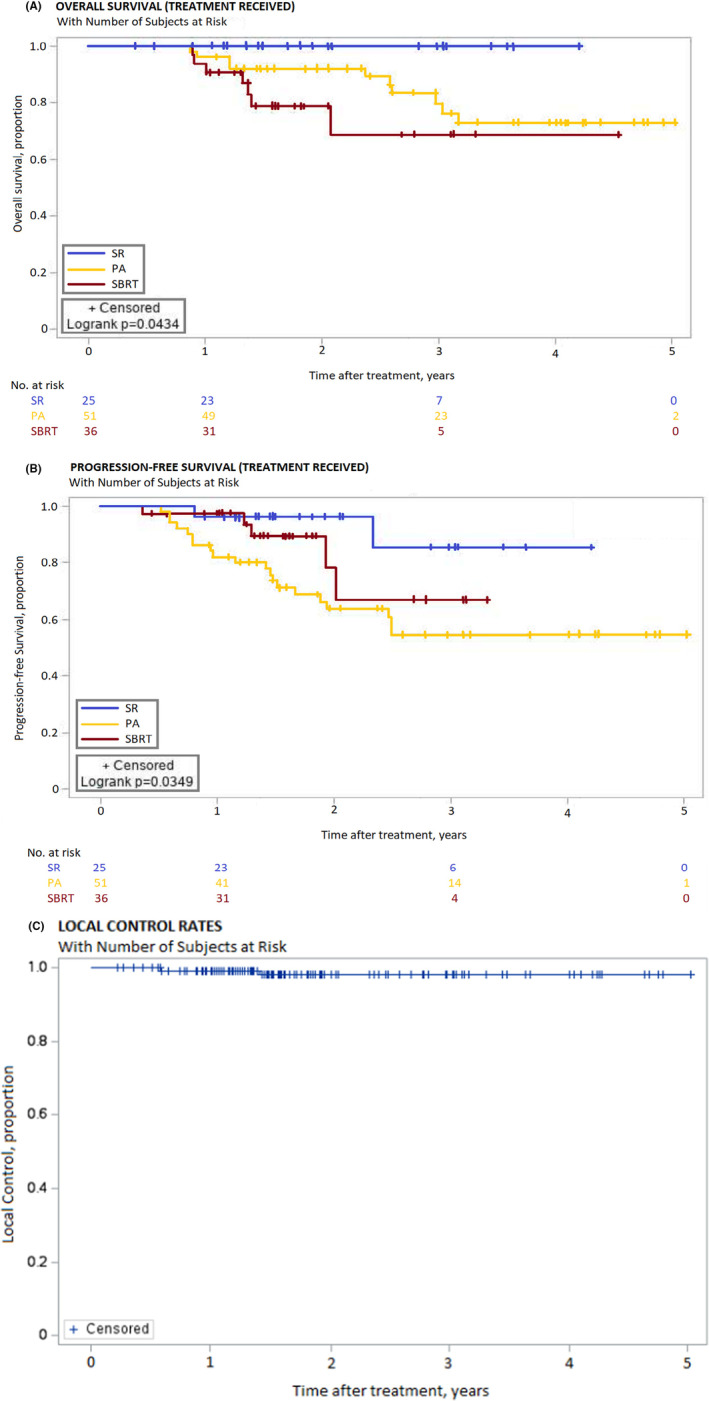
Kaplan–Meier Curves for (A) Overall survival, (B) Progression‐free survival, (C) Local control.

The median follow‐up of all patients (alive or deceased) at the closeout date was 23.1 months (range, 3–60). The 3‐year OS for the entire study cohort was 80%. Patients who underwent SR had significantly better 3‐year OS (100%) compared to SBRT (69%) and PA (80%) (*p* = 0.04). (Figure 2A) There was no significant difference in 3‐year OS between the SBRT and PA cohorts (HR 0.54, 95% CI 0.2–1.48, *p* = 0.23).

At the study close‐out date, 95 patients (73.1%) were alive or had not been transplanted. There were 17 deaths in total. There were no deaths in the SR cohort. In the SBRT cohort, there were seven deaths, including one cancer‐related death, three liver failure‐related and three due to other causes. In the PA cohort, there were 10 deaths, including five cancer‐related deaths, one liver failure‐related and four due to other causes. Four patients (3.6%) were transplanted, three for management of HCC with disease progression and one for decompensated cirrhosis.

### 3.3 Progression‐free survival (PFS) and local control (LC)

The 3‐year PFS was significantly different between the three treatments (*p* = 0.04). (Figure 2B) The 3‐year PFS for SR was 85%, SBRT was 67% and PA was 55%. Patients treated by SR had a decreased risk of disease progression compared to the PA cohort (HR 0.21, 95% CI 0.05–0.90, *p* = 0.04). There was no significant difference in PFS between the SBRT and PA cohorts (HR 0.48, 95% CI 0.18–1.30, *p* = 0.15).

Disease progression occurred in 27 patients (24%). Extrahepatic disease progression occurred in four (8%) of patients, two of whom had local intrahepatic disease progression (treatment failure). Out of field, intrahepatic disease progression occurred in 23 patients (20.5%) and 13 of these patients had indeterminate, LiRADS‐3 lesions at the time of diagnosis and MDM. A higher proportion of patients in the PA group (37.3%) had LiRADS‐3 lesions compared to the SR (16%, *p* = 0.11) and SBRT groups (13.9%, *p* = 0.03). Upon disease progression, 25 of the 27 patients had subsequent treatment as per MDM recommendations.

In the PA group, two patients (4%) developed local recurrence, 16 (31%) out of field, intrahepatic disease progression and four (8%) extrahepatic disease progression (including the two patients with local recurrence). Of the 16 patients with out of field, intrahepatic disease progression, 10 patients received curative intent therapy with either SBRT (*n* = 5), PA (*n* = 3) or LT (*n* = 2). Of the four patients with extrahepatic disease, systemic therapy was provided to three patients.

There were no cases of local recurrence or extrahepatic disease progression in the SBRT or SR cohorts. There were five (14%) and two (8%) cases of out of field, intrahepatic disease progression in the SBRT and SR cohorts, respectively. In the SBRT group, three patients were treated with TACE, one with PA and one had a LT. In the SR group, one patient received TACE and the other SBRT.

### 3.4 Factors associated with survival outcomes

On univariate analysis, aetiology of liver disease (non‐viral vs viral), older age, performance status (CCI and ECOG) and worse hepatic function (CP, MELD, ALBI) were associated with inferior OS (Table 2). None of these factors remained significant on multivariate analysis (MVA) (Table 2). Univariate analysis demonstrated that the presence of a LiRADS‐3 lesion was associated with inferior PFS (HR 4.2, 95% CI 2.0–8.94, *p* < 0.01) and this remained significant on MVA (Table 3).

**TABLE 2:** Factors associated with overall survival.

	Univariate analysis		Multivariate analysis	
Characteristic	HR (95% CI)	*p*	HR (95% CI)	*p*
Age	1.07 (1.02–1.12)	<0.05	1.06 (0.97–1.15)	0.18
Gender (M)	0.60 (0.27–1.32)	0.21	–	–
Aetiology (viral vs non‐viral)	0.38 (0.18–0.82)	<0.05	0.71 (0.14–3.65)	0.68
CCI (each single point rise)	1.44 (1.19–1.74)	<.05	1.10 (0.80–1.52)	0.54
ECOG (each single point rise)	2.33 (1.40–3.90)	<0.05	1.19 (0.77–4.59)	0.16
Diagnosis on surveillance (Y)	0.97 (0.40–2.41)	0.96	–	–
Cirrhosis (Y)	N/A	0.99	–	–
CP (each single point rise)	1.98 (1.28–3.07)	<0.05	1.02 (0.22–4.71)	0.98
MELD (each single point rise)	1.12 (1.01–1.23)	<0.05	–	–
ALBI (each single point rise)	3.68 (1.87–7.24)	<0.05	2.3 (0.68–7.79)	0.18
Presence of PHT (Y)	0.72 (0.34–1.50)	0.38	–	–
Prior decompensation (Y)	0.51 (0.21–1.25)	0.14	–	–
Presence of ascites (Y)	0.59 (0.21–1.71)	0.33	–	–
Size (each mm increase)	1.04 (0.97 0 1.13)	0.27	–	–
BCLC‐A (in comparison to BCLC‐0)	1.62 (0.73–3.60)	0.23	0.57 (0.15–2.11)	0.28
Treatment Received	N/A	N/A	1.88 (0.61–5.84)	0.27

**TABLE 3:** Factors associated with progression‐free survival.

	Univariate analysis		Multivariate analysis	
Characteristic	HR (95% CI)	*p*	HR (95% CI)	*p*
Age	0.99 (0.95–1.03)	0.68	1.04 (0.99–1.12)	0.11
Gender (M)	1.81 (0.62–5.23)	0.28	–	–
Aetiology (viral vs non‐viral)	1.66 (0.70–3.94)	0.25	3.76 (0.96–14.79)	0.06
CCI (each single point rise)	0.93 (0.76–1.15)	0.52	0.90 (0.69–1.18)	0.44
ECOG (each single point rise)	0.72 (0.31–1.67)	0.44	1.37 (0.43–4.34)	0.59
Diagnosis on surveillance (Y)	1.14 (0.46–2.82)	0.78	–	–
Cirrhosis (Y)	N/A	0.99	–	–
CP (each single point rise)	1.30 (0.80–2.11)	0.28	1.78 (0.32–9.75)	0.51
MELD (each single point rise)	1.06 (0.94–1.18)	0.34	1.06 (0.89–1.27)	0.48
ALBI (each single point rise)	1.55 (0.78–3.06)	0.21	2.07 (0.79–5.45)	0.14
Presence of PHT (Y)	0.88 (0.41–1.89)	0.75	–	–
Prior decompensation (Y)	0.81 (0.28–2.33)	0.69	–	–
Presence of ascites (Y)	2.36 (0.32–17.41)	0.40	–	–
Size (each mm increase)	0.96 (0.89–1.04)	0.33	–	–
BCLC‐A (in comparison to BCLC‐0)	0.70 (0.33–1.50)	0.36	0.32 (0.12–0.89)	0.03
Treatment received	N/A	N/A	1.43 (0.66–3.11)	0.36
Presence of indeterminate lesion	4.18 (1.96–8.95)	<0.01	7.29 (2.79–19.06)	<0.01

### 3.5 Adverse events

Treatment‐related clinical toxicities or complications were described in Table 4. There were no deaths within 3 months after any of the treatments. No patient experienced a ≥1‐point change in CP within 3 months post treatment. Beyond 3 months post treatment, four patients in the SBRT cohort had a >1 point change in CP. Three of these patients had intercurrent illnesses including spontaneous bacterial peritonitis in two patients and bone fracture in one patient. The other patient had out of field HCC progression with rupture. The patient in the PA cohort that had a >1 point change in CP was evaluated at the time of an intercurrent illness with spontaneous bacterial peritonitis.

**TABLE 4:** Treatment‐related toxicity or complications.

	SR (*n* = 25)	PA (*n* = 51)	SBRT (*n* = 36)
Grade 0	21	44	18
Grade 1 or 2	Nausea/vomiting, *n* = 2	Pain, *n* = 1 Fever, *n* = 1 Post embolization syndrome, *n* = 3 Myasthenia gravis exacerbation, *n* = 1 Pneumothorax, *n* = 1	Nausea/vomiting, *n* = 6 Fatigue, *n* = 11 Pain, *n* = 1
Grade 3 or 4	Abscess requiring percutaneous drainage, *n* = 1 Respiratory failure due to hospital acquired pneumonia requiring ICU, *n* = 1	0	0

## 4 DISCUSSION

In a favourable prognostic cohort of patients with a new diagnosis of BCLC early‐stage and solitary HCC up to 3 cm with preserved liver function, we found SBRT to have comparable LC rates to SR and PA and in non‐surgical patients comparable 3‐year PFS and OS to PA. Local control was excellent across all treatment modalities with only two local failures, both in the PA cohort. In the entire cohort, local control at 1‐year and 3‐year were 99% and 98%, respectively. Patients who underwent surgery had significantly better 3‐year OS and PFS compared to the SBRT and PA cohorts (*p* = 0.04), however, there was no significant difference in 3‐year OS or PFS between the SBRT and PA groups. These differences in OS and PFS will be heavily influenced by selection bias, in particular the PA group had a higher proportion of indeterminate LiRADS‐3 lesions at baseline with some subsequent early progression events whilst the SBRT group were older and frailer.

PA is the standard curative intent therapy for BCLC early‐stage small (≤3 cm) solitary HCC in patients deemed not to be suitable surgical candidates. However, the efficacy of PA is limited by tumour size (≤3 cm) and location.^7^, ^12^ Non‐surgical patients deemed unsuitable for PA may undergo treatment stage migration and be offered palliative intent therapies such as TACE. In some studies more than 40% of patients with BCLC early‐stage solitary ≤3 cm HCC underwent treatment stage migration as they were unsuitable for PA.^5^, ^13^ In our study, over 30% of patients with a new diagnosis of a solitary HCC up to 3 cm in size were not suitable for PA or SR. North American clinical guidelines have recently recommended SBRT as a potential liver directed therapeutic option for these patients.^14^, ^15^ With the inclusion of SBRT as an alternative first‐line treatment option within our MDM, 90% of the original 123 patients identified with BCLC early‐stage solitary ≤3 cm HCC received a curative intent first‐line therapy and only 5% received treatment stage migration therapy with TACE. The remainder were either unfit for any treatment or declined therapy.

Recent prospective studies from Japan and France have evaluated SBRT in the treatment naïve, early‐stage, solitary HCC setting. Kimura et al. evaluated 36 patients with treatment naïve, solitary HCCs up to 5 cm and CP of ≤B7 who were unsuitable for surgical management or PA and underwent SBRT to 40 Gy in five fractions.^16^ The median size of the treated HCC was 2.3 cm. They reported 2‐year LC rates of 90%, recurrence free survival of 59% and OS of 84%.^16^ There were no grade 3–5 treatment‐related clinical toxicities within 3 months post treatment.^16^ After 3 months, Kimura et al. reported four patients (11%) had grade 3 treatment‐related clinical toxicities, three of which were gastro‐intestinal toxicities and 12 patients (34.3%) were observed to have a >1 point change in CP.^16^ In a prospective multi‐centre French study, (Durand‐Labrunie et al) evaluated 43 patients who were treatment naïve, had a solitary HCC up to 6 cm and underwent SBRT to 45 Gy in three fractions.^17^ The authors reported two‐year LC rates of 94%, PFS of 48% and OS of 69% following SBRT.^17^ Grade ≥3 adverse events were gastro‐intestinal related and observed in 5% of patients.^17^ In our SBRT cohort, there were no treatment‐related grade 3–5 clinical toxicities, likely a reflection of our conservative dose constraints, advanced motion management using a predominant breath hold work flow and on‐treatment soft tissue verification imaging.

In Australia, we have observed an increased utilisation of SBRT in the management of HCC including as a first‐line, curative intent treatment.^9^ An Australia multi‐centre retrospective review reported outcomes following curative intent SBRT in 68 patients with treatment naïve BCLC early‐stage, solitary HCC up to 5 cm with compensated CP A cirrhosis.^18^ The two‐year freedom from local progression was 94%, PFS was 60% and OS was 88%.^18^ Grade 2 clinically significant and treatment‐related toxicities were observed in 13.2% patients and there were no grade 3–5 treatment‐related clinical toxicities.^18^

During the COVID‐19 pandemic and in the current health environment with hospital bed pressures, an outpatient treatment modality is invaluable in providing patients with cancer directed treatment within an appropriate time frame. SBRT was typically administered over three to five fractions in an outpatient setting. In our study, the median inpatient hospital stay was 6 days for SR and 3 days for PA. In recent years, there has been increasing focus on specific indicators of quality care in the management of patients with HCC. For HCC, one quality indicator determined by an Australian multidisciplinary task force was the ability to commence treatment within 4 weeks of decision to treat at time of MDM.^19^ The mean time from MDM to SBRT delivery was significantly shorter at 32.8 days, compared to 51.5 days for PA and 61.9 days for SR (*p* < 0.05) over the time period of this study. Another advantage of SBRT is that it can be delivered to HCC in locations that are not technically accessible by PA. Finally, as demonstrated in our cohort, SBRT offers the potential of curative therapy with minimal toxicity and morbidity to the older, frailer patient with more co‐morbidities and advanced liver disease.

This study was limited by its retrospective, single‐centre nature and the limited follow‐up period. On the univariate analysis several factors were associated with reductions in OS, however, none were significant when assessed on multivariate analysis. This is likely due to the small number of patients in each cohort and highlights the need for multi‐centre studies with greater patient numbers. Despite this, our study provides valuable real‐world data in the management of BCLC early‐stage, solitary HCCs up to 3 cm when considering liver directed therapeutic options and avoiding treatment stage migration. The inclusion of SBRT within our MDM as an additional treatment option resulted in 90% of the cohort receiving a curative intent first‐line therapy. We believe this approach also allowed the optimal deployment of each treatment modality with only two local failures, both in the PA cohort. Additional comparative randomised evidence is required to further define the role of SBRT in the first‐line setting. TROG 21.07, a multicenter, randomised controlled trial for patients with newly diagnosed BCLC stage 0/A, solitary HCC up to 8 cm and unsuitable for SR or LT is currently enrolling patients across 16 centres in Australia. (ACTRN1262100144875) This trial is comparing SBRT with PA for HCCs up to 3 cm, and for HCCs more than 3 cm or unsuitable for PA, the comparison is between SBRT and the investigator's choice of transarterial therapies with or without PA. The primary endpoint is 2‐year local control with secondary endpoints including PFS and OS, toxicity and quality of life measures.

## 5 CONCLUSIONS

In patients with treatment naïve, early‐stage, solitary HCC up to 3 cm, use of SBRT was associated with comparable local control, PFS and OS outcomes to PA. SBRT should be considered a curative intent therapy to avoid treatment stage migration in this favourable prognostic cohort of patients. Ongoing trials will further define the role of SBRT in early‐stage HCC.

## AUTHOR CONTRIBUTIONS

**A. M. Maher:** Conceptualization (supporting); data curation (lead); formal analysis (supporting); investigation (equal); methodology (supporting); writing – original draft (lead); writing – review and editing (equal). **M. Shanker:** Data curation (equal); formal analysis (lead); investigation (equal); methodology (equal); writing – review and editing (supporting). **H. Y. H. Liu:** Conceptualization (equal); formal analysis (equal); methodology (equal); supervision (supporting); writing – review and editing (equal). **Y. Lee:** Writing – review and editing (supporting). **D. Leggett:** Writing – review and editing (supporting). **P. Hodgkinson:** Writing – review and editing (supporting). **D. Pryor:** Writing – review and editing (supporting). **K. A. Stuart:** Conceptualization (equal); formal analysis (equal); methodology (lead); supervision (lead); writing – original draft (supporting); writing – review and editing (lead).

## FUNDING INFORMATION

None.

## CONFLICT OF INTEREST STATEMENT

All authors had substantial contribution to the conception and analysis of the work, drafting/revising of the manuscript and final approval for publication.

## Data Availability

Author elects to not share data.
